# Wireless Health Data Exchange for Home Healthcare Monitoring Systems

**DOI:** 10.3390/s100403243

**Published:** 2010-04-01

**Authors:** Malrey Lee, Thomas M. Gatton

**Affiliations:** 1 Center for Advanced Image and Information Technology, School of Electronics & Information Engineering, ChonBuk National University, 664-14, 1Ga, DeokJin-Dong, JeonJu, ChonBuk, 561-756, Korea; 2 The School of Engineering and Technology, National University, 11255 North Torrey Pines Road, La Jolla, CA 92037, USA; E-Mail: tgatton@nu.edu

**Keywords:** home health care monitoring, HL7, IEEE1451, data exchange, wireless sensor network

## Abstract

Ubiquitous home healthcare systems have been playing an increasingly significant role in the treatment and management of chronic diseases, such as diabetes and hypertension, but progress has been hampered by the lack of standardization in the exchange of medical health care information. In an effort to establish standardization, this paper proposes a home healthcare monitoring system data exchange scheme between the HL7 standard and the IEEE1451 standard. IEEE1451 is a standard for special sensor networks, such as industrial control and smart homes, and defines a suite of interfaces that communicate among heterogeneous networks. HL7 is the standard for medical information exchange among medical organizations and medical personnel. While it provides a flexible data exchange in health care domains, it does not provide for data exchange with sensors. Thus, it is necessary to develop a data exchange schema to convert data between the HL7 and the IEEE1451 standard. This paper proposes a schema that can exchange data between HL7 devices and the monitoring device, and conforms to the IEEE 1451 standard. The experimental results and conclusions of this approach are presented and show the feasibility of the proposed exchange schema.

## 1. Introduction

Because rapidly increasing populations have overloaded healthcare systems, many countries have been focusing attention on potential computer-based solutions for provision of home healthcare to an aging population. Ubiquitous healthcare (U-healthcare) systems can provide a convenient and economical way to provide healthcare for patients who have been diagnosed with a chronic disease, such as diabetes and hypertension. In the home healthcare scenario, sensors are distributed in residences and placed on the patient’s body to transmit sensor data to the appropriate medical organization. The collection, transmission and processing of this physiological data requires a simple, wireless sensor network and a personal digital device, such as a PDA [1–4].

**Figure 1.**
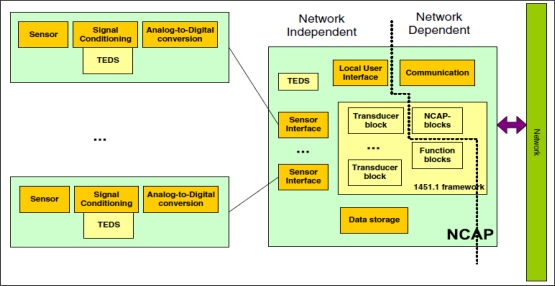
Sensor node and NCAP structure.

The IEEE 1451 standard is comprised of several standards and defines an Application Program Interface (API) for applications that provides communications between smart transducers, such as sensors and actuators. The goal of these standards is to provide an interface schema that allows connections between different devices and heterogeneous networks. The IEEE 1451.0 standard defines a set of functionalities for the IEEE 1451 standard smart transducer interface, and it is independent of physical communication media. The Transducer Electronic Data Sheet (TEDS) is defined by IEEE 1451.0, and is the fundamental specification of the family of IEEE 1451 standards. TEDS resembles the BIOS information that is stored in COMS, in personal computer systems, and it describes the physical information about the transducer, such as battery and data unit information, and a Network Capable Application Processor (NCAP), such as a PDA that can process data from sensors, based on information that is stored in sensor nodes. The Transducer Interface Module (TIM) is the interface module that provides sensor data and the state of sensor nodes to the NCAP. The NCAP can determine the communication speed, channel number and data format, which is stored in sensor nodes from TIM, through query to the TEDS. The IEEE 1451 standards are shown in Figure 1.

Health Level 7 (HL 7) is the most popular standard for medical information exchange, and is approved by ANSI. The goal of the HL7 standard is to exchange information among clinics, insurance companies, and health management organizations, based on text messages. The most important concepts of HL7 are triggers, trigger events, and messages. Triggers are used to listen for trigger events, such as “the patient is going to arrive”. The trigger event is detected as a trigger, and directs the application to prepare specific documents, such as the clinical record, financial account, and insurance information of a patient. HL7 documents and messages are text-based information, and use the XML file structure to store information. The message structure follows the most current HL7 v3 standard when using XML to encapsulate, thereby providing a flexible way to exchange information. The HL7 messages are easy to encode and, because the information is text-based and has mature technology for encryption, provide satisfactory reliability in the application layer [5,6].

Because the HL7 standard does not yet support mobile device application, the development of middleware to transfer messages and commands between the HL7 and IEEE 1451 standards is crucial and the primary focus of this research. The IEEE 1451 is preferable over the IEEE P1073 standard for several reasons. The IEEE P1073 family is for professional medical device communication, and standard IEEE P1073.1.3.6 and P1073.1.3.7 describe electrocardiogram (ECG) and blood pressure data communication protocols [7,8]. These standards are not easy to implement for other medical device communication requirements on mobile devices. Also, because the IEEE P1073 is still in development, the details of that standard could be modified in the future, requiring implementation modification. The IEEE 1451.0 standard has been approved by IEEE, and is stable for any future implementations. Finally, the TEDS in the IEEE 1451 standard supports user-defined data sheets, allowing flexible user-defined TEDS with XML or other text format. Thus, use of the IEEE 1451 can reduce implementation workload requirements for communication and data exchange with HL7 applications. This paper proposes a data exchange schema that can exchange medical data between an x86 personal computer and an embedded device over a Personal Digital Assistant (PDA) device. HL 7 format data is applied on the personal computer and IEEE 1451 format data is applied on the embedded device.

This is the fundamental motivation and basic background for this research. Section 2 now provides the background material and describes related work in comparison to this approach. Section 3 introduces the proposed middleware structure and its basic operation, and Section 4 presents the system implementation. Finally, Section 5 provides the experimental results and the conclusions and recommendations are given in Section 6.

## 2. Background and Related Work

Many researchers have proposed U-healthcare monitoring system architectures. Black, *et al*. proposed a scenario system that is called “pervasive computing in health care” [9], which integrates with enterprise applications, including software, hardware, databases, standards and life cycle. This work considered the issues of implementation, such as network media, network effective ranges, the methods of connection among PDA and hospital, system updating and compatibilities, in a real environment. Kim and Lim proposed integrating IEEE 1451 and HL7 information [10], with a PDA that collected data from the patient’s body sensors, and communicated with a personal computer through networks. However, [9] was a prototype healthcare system, and requires further development to address technical areas, such as database, network protocols and hardware, for actual implementation [10]. Although this work integrated IEEE 1451 and HL7 data, commercial sensor devices only conform to one standard, due to production cost consideration, and integration of data both in IEEE 1451 and HL7 are difficult to apply in real applications.

This proposal focuses on the data exchange between devices which conform to the IEEE 1451 and HL 7 standards. The main goals of this proposal are:

- Define the Healthcare system architecture which conforms to the HL 7 standard.
- Define the fundamental structure on the end-user side, such as PDA sensor data collection and communications.

The research approach concentrates on the implementation of a healthcare monitoring system that consists of monitor data collection and a display system. It differs from the previously described approaches, because it is not scenario-based and it concentrates on monitoring data exchanges. Further, the patient sensor and medical information shown in the monitoring center resolves the difference between existing standards. This approach provides an improved solution, as compared to previous approaches [9,10], and provides a system that is feasible in a real-world implementation environment.

## 3. Healthcare Data Monitoring System

**Figure 2.**
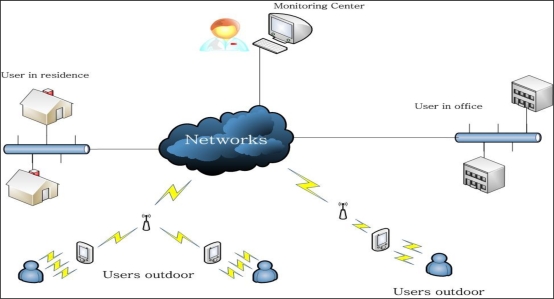
The health monitoring system deployment architecture.

The proposed system remotely monitors the states of patients and relays electrocardiogram (ECG), temperature, glucose, and/or other types of data. Patients are required to wear the appropriate sensor(s), have a mobile device, such as a PDA, and be in an area with suitable, wireless network access. Generally, sensors cannot communicate directly with wireless networks, due to prohibitive production costs, and a sensor needs hardware and software that provide the requisite communication. In the proposed configuration, the sensors are connected to a closely located PDA through a wireless connection, such as the Bluetooth connection, as shown in Figure 2. The PDA can connect to a wireless Access Point (AP) or router, thus providing the communication channel with the monitoring center. The sensor can be an RFID tag on the patient which provides the exchange of data with the PDA through a wired connection or wireless Bluetooth. The user should register with monitoring center to acquire the necessary preliminary information, recording personal information, present illness and illness history, etc., and be assigned a unique medical identification tag. The monitoring center can then provide suitable security and verification of the user identity for remote access to the server. The network can be an xDSL, optical fiber or other access type provided by the Internet Server Provider (ISP), and the wired access cable can connect to the wireless router. While this type of wireless access can be easily distributed in a residence or office, the wireless router cannot provide seamless roaming. This is because the PDA needs to re-access and re-require a new IP address while roaming between various wireless areas. The solution is to provide an access point (AP), which are wired connections to a switch hub or router, and allow the PDA to connect to access points though this wireless connection and allow PDA roaming. This proposal is focused on monitoring and data transmission and uses the wireless router as the transmission media, due to equipment restrictions.

The monitoring center can selectively receive the user’s sensor data when some of the data is not necessary and determine the communication connection necessary to monitor patient data. The monitoring center can send commands to the PDA, and receive PDA responses to those commands, as required.

### 3.1. Healthcare Monitoring System Architecture

**Figure 3.**
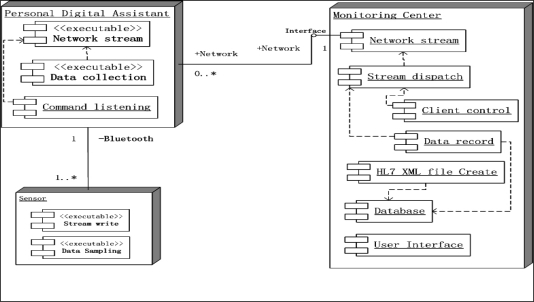
Healthcare monitoring system architecture.

The healthcare monitoring system adopts the Client/Server (C/S) structure, as shown in Figure 3, where the clients conform to the IEEE 1451 standard. IEEE 1451 is a set of smart transducer interface standards and the IEEE 1451.0 sub-standard is applied in this system. The dot 0 defines the commands and common TEDS, and other sub-standards follow the dot 0 standard. The patient information is recorded on the TEDS client side, and the server side records patient information, such as the user id and personal information, and verifies data from TEDS when verifying client access. The application program on the PDA contains the network stream class and data collection class, as shown in Figure 3. The data collection class collects data from the sensor(s) and the network stream class sends the data stream to the server. The functions of the client and server are:

***Common function:***

- Network stream: Stream data send and receive class - PDA can only connect to one monitoring center, but the monitoring center can have none or multiple PDA connections.

***Sensor functions:***

- Stream write: The class sending data to PDA.
- Data sampling: Sampling data using physical sensors.

***PDA functions:***

- Command listening: Listening to the network and responding to server with relayed commands. This function is the relay for the network stream class.
- Data collection: Collects sampling data from sensors, and sends it to the server, according to server requirements.

***Monitoring Center:***

- Stream dispatch: The monitoring center receives sampling data and response information, and the stream dispatch class transmits the data to corresponding process class.
- Client control: Command sending class. Medical personnel select the information to monitor, translated into the corresponding commands and transmitted to the PDA.
- Data record: Records sampling data from PDAs.
- HL7 XML file create: Create XML files for the patient conforming to HL 7 v2.5 standard. XML document records the personal information, symptoms, present illness, history illness, and necessary sampling data. Each user can have one XML file.
- Database: Records the history sampling data of the patients.

#### 3.1.1. Personal Digital Assistant

The main function of the PDA is to transmit the required data to the server, and, in the proposed system, simulate the sensors. The data in TEDS is the basic information that records the hardware performance and the property of sampling data. The proposed system contains the Meta-TEDS and the Transducer Channel TEDS. The meta-TEDS provides the worst-case timing parameters for the PDA application, and also sets a time-out value to indicate the maximum server response wait time, before the data is discarded. The remaining data of meta-TEDS defines the relationships of the Transducer Channels [11]. Transducer Channel TEDS provides detailed information about a specific sensor, and describes the physical property and unit of the sampling data that is being measured. Both TEDSs have a common area called the TEDS header, as shown in Table 1.

Table 1 demonstrates a non-standard and modified TDES table header, with a cleaner structure and easier programming. The size of each field in header occupies 1 byte, thus, the tuple length field contains 255 bytes, at most, and cases larger than that are not considered in this TEDS.

Table 2 is a partial list of Meta-TEDS and TransducerChannel TEDS, to illustrate the data fields that are useful in the proposed system:

The UUID field in Table 2 is the unique ID for specific sensor devices and users, where the unique medical identification for a specific user is a part of UUID. Note that each sensor device maintains private TEDSs, which do not interfere with TEDSs in other sensor devices. The medical identification recorded in UUID for a specific user in each sensor device can be independent of the mobile device. The user can use arbitrary mobile devices, which can support Bluetooth and network server access, allowing more flexibility to the user. The OholeOff field is the time interval for operational time-out in seconds, and the lack of a receipt from a command after the time-out is interpreted as a failed operation. The TestTime field indicates that the time interval to self-test, and report any hardware fault to the server, in seconds. The MaxChan field indicates the maximum number of communication channels that can be established between the sensor and server, and is greater than or equal to one. The ChanType field in Table 3 is always 0 in the proposed system. The UnitType field enumerates the unit type of the sampled data, such as millivolts (mV), centigrade (C) or Fahrenheit (F). The DatModel indicates the type of sampling data, such as ECG or temperature data. The data type can be 32 bit floating point or 64-bit double precision floating point, integer, long integer or a bit sequence for specific sampling data. The SigBits indicate the most significant bit for the specific data type. The Speriod field indicates the time interval between two sampling data. This field is variable for different sampling type, for temperature data can be sampled per 6 hours, and the ECG data can be sampled every 3 milliseconds.

TEDS defines the physical profile of the sensor hardware and the properties of the sampled data. TEDS is a read only information table, and this information is transmitted at the beginning of transmission, before the sampling data transmission, and is not required during transmission. More details of TEDS can found by referring to the IEEE 1451.0 standard.

**Figure 4.**
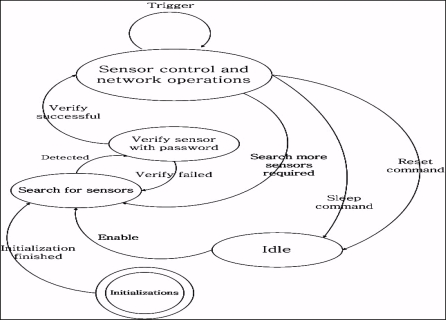
Mobile device running.

The sensor devices on the user side are independent of the mobile device type. The global unique medical identification for a specific user is recorded in the UUID field in Meta-TEDS, as shown in Table 2, of each sensor, and it is the only identification that the server uses for recognition, as shown in Figure 4.

A password could be assigned by user when first time register to or purchase from medical organizations, and this password is needed when the sensor connect to mobile device. The mobile device functions between the sensor(s) and server transmissions. The mobile device searches the sensors when it launches and connects to the server when a sensor is detected and authenticated. The authentication process of the mobile device fails when multiple sensors are detected and the medical identification in any UUID field is different. Authentication failure can also occur when the server password verification fails. The mobile device work states shown in Figure 4 will change to the “search for sensors” state, after initialization. It remains in this state until some sensors are detected. It then changes to the “Verify sensor with password” state and asks for the sensor password and medical identification number. If the server successfully verifies, the mobile device state changes to “Sensor control and network operation”. The mobile device forwards the data stream coming from the sensor to the server. The mobile device listens to the network for commands and, if any trigger commands are received, such as “Stop ECG data sending”, the mobile device will control the related sensor, but still keep connected to the sensor. The “Idle” state is for devices self-testing, if any abnormalities are detected by the server.

#### 3.1.2. The Monitoring Center

**Figure 5.**
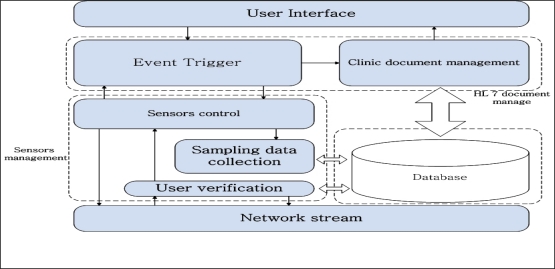
Monitoring software architecture.

The HL 7 standard is an event-driven framework and essentially the same as event-driven processing in Win32 programs. The monitoring system generates an “Add new” event when a user first registers with the monitoring center. This event could lead to several actions, such as “Create a clinic document for user”, “Create a bill account for user”, or a “user information request” event generated by medical personnel. Subsequently a “send request info message” or “receive request info message” is sent. Applications conforming to the HL 7 standard rely on the seventh, or application layer, of the OSI network architecture. Following the HL 7 standard, the monitoring center design does not consider the physical connection method, but defines a series of handshake protocols at the application layer. The monitoring center software consists of a sensors management and an HL 7 document management tier, as shown in Figure 5.

##### A. Sensors management

The sensors management level controls the sensors with a series of commands. The protocol of the underlying network is TCP based, because the information for user verification and sampling data are security sensitive, and TCP protocol provides a more reliable connection than UDP. Additionally, the connection link can use encryption, such as Secure Sockets Layer (SSL). All user information, including the medical identification and password, is stored in the database. The sensors management module authenticates the user connection request and compares the request data with the register data in the database. If verification fails, information access is denied. Otherwise, control passes from the user medical identification tier to the sensor control module and the user is notified of authentication success. The sensors control module now directly controls the mobile device and queries for connection information from mobile device. The number of connection links and sampling data properties are requested from the mobile device and sent to the server. The server then establishes new connection links on specific ports and the mobile device sends the sampling data directly to the server through the new links. This sampling data is recorded in the database. Both the sensor control and user verification module maintain a private user queue to contain the multiple user requests, so that the two queues do not interfere with each other. The user verification module queue is a very dynamic queue, as the requesting user is removed, regardless of whether authentication failed or succeeded. Correspondingly, the queue in sensor control module does not change as much, because the user is added to the queue when authentication is success and removed when the user disconnects from the server.

##### B. HL 7 document management

The document management module is between Graphical User Interface (GUI) and sensors control, through the event trigger handling, and it is the main control module of the software. The majority of the events are created by medical personnel through the GUI. When a user name is clicked in the user list, the GUI generates a user view event, and the event trigger module responds to this event and locates the user information by invoking the clinic document management module. The main function of clinic document management module is to create, revise, view, and delete user information. A clinic document is formed as an XML file conforming to the CDA standard of HL7. The clinic document records all of the personal information, such as name, gender, age, home address, symptoms, present illness, illness history, images, etc. All data that appears in the XML file has a copy in the database, to prevent deletion of the XML file by mistake. Either an XML file, with sampling data and a user list, is saved on the hard disk when the application is closed, or there is a database record of the information. The real-time sampling data stream, such as ECG data, is drawn as images, which can be saved as determined so by a medical person. This significantly reduces data storage requirements, as a representative sample is sufficient.

### 3.2. Communication Protocols

The communication protocols define the exchange criteria between the sensor and mobile device, the mobile device and server, and the sensor and the server. The protocol between sensor and mobile device is referred to as the Service Discovery Protocol (SDP), which is a protocol in the Bluetooth standard. The sensor has little difference from a Bluetooth device, so the SDP requires only a few modifications to be suitable for the proposed approach. Since the SDP of Bluetooth works at the link layer, and the protocol between the mobile device and the sensor occurs after successful linkage, the change only affects the application layer. The service discovery is the only processes where the mobile device connects to the sensor. The successor protocols are used for control commands and data exchanges between server and mobile device, or sensors. The control commands are listed in Table 4.

Mobile devices maintain one channel for each sensor, and each channel is used for both commands and sampling data transmission. The server can maintain several channels with one mobile device, one channel for command transmissions, and other channels for sampling data transmission. The command channel is the default channel that is established when the connection request is approved. Once the connection is approved, the server requires more information about sampling data transmission, such as information stored in the TEDS for each sensor. Command 8, “sr_QueryTEDS”, is the first command that the server sends to the sensor through a mobile device. This command obtains the category of TEDSs that the sensor supports, assigns a sensor support Meta-TEDS and TransducerChannel TEDS, and returns a message to inform the server it can Meta and TransducerChannel TEDSs. The server then knows the amount of TEDSs that can be read. The capacity of the sampling data channel depends on how much sampling data each sensor can transmit in the private channel, and in a common command channel. The channel is essentially the ports, eg., assuming an IP address of 192.168.0.1, the command channel can be 192.168.0.1 at port 1024, and one of the sampling data channel can be 192.168.0.1 at port 1025.

#### 3.2.1. Protocols between a Mobile Device and a Sensor

**Figure 6.**
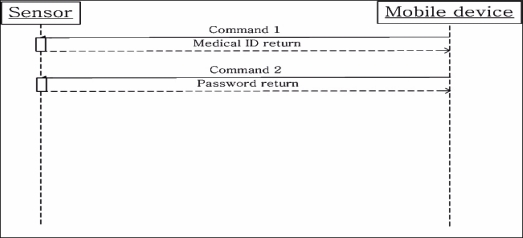
Protocol between mobile device and sensor.

The protocol between a mobile device and a sensor is shown in Figure 6, below.

Command 1, as described in Table 4, transmits from the mobile device to a sensor after the connection is established in the link layer. The mobile device requests medical identification from the sensor and after the sensor returns it, the mobile device asks for the password from sensor. The program which is running on mobile device uses a decryption algorithm for the user ID and password authentication, and upon successful authentication, the mobile device connects to the server through the previously set IP address.

#### 3.2.2. Protocol between the Sensor, Mobile Device and Server

**Figure 7.**
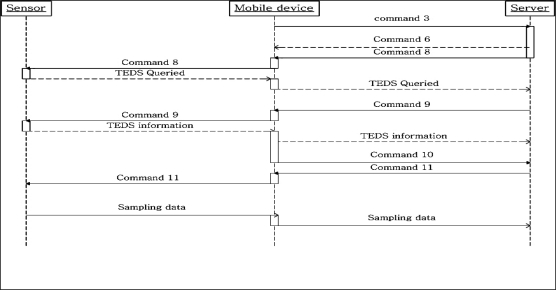
The protocol between the sensor, mobile device and server.

Once the mobile device successfully authenticates the user, it connects to server, as shown in Figure 7. The mobile device sends Command 3, md_ConntReq, to the server requesting connection, and the server returns Command 6, sr_ConntAprv, to the mobile device, if the server is not overload. The server then prepares for mobile device connection, but queries for the memory size needed for the TEDS information. The server sends Command 8, sr_QueryTEDS, to obtain this information and the mobile device forwards the command to the sensor, and returns the sensor response to the server. The server allocates sufficient memory and requests the sensor to transmit the sampling data property using Command 9, sr_ReadTEDS. The sampling data property is sent back to the server through the mobile device the server assigns a new channel for sampling data transmission with Command 10, sr_ChanPara. The server now has the sampling data property and channel information, and request the mobile device to send the sampling data using a new data channel, using Command 11, sr_InfoCorm. The port number of the new data channel is recorded in sr_InfoCorm as a parameter, and the mobile device begins collect sampling data from sensor and forwarding it to the server with the new channel.

## 4. Implementation

The proposed health monitoring system is implemented with hardware and software that is widely available, cost effective and easily supported. The monitoring software runs on personal computer with x86 architecture, and the client program on the user side is running on a PDA with windows mobile 5 (WM 5). The sensor is simulated on the PDA, and TEDS and sampling data are stored as a file on the flash memory. The test environment specifications are listed below in Table 5.

### 4.1. Sampling Data and Program Class

**Figure 8.**
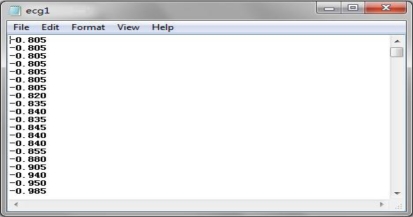
ECG data in text format.

The two types of sampling data used in this experiment are ECG data and body temperature. A normal ECG data is a continuous stream of data with a sample rate of several milliseconds, thereby generating a large dataset within several seconds. The body temperature is sampled several times per day, consisting of three times in the morning, afternoon and night respectively. The ECG sampling data is adopted from PhysioBank’s 123rd record with MLII signals in the MIT-BIT Arrhythmia Database. The data is contained using text formatting, as shown in Figure 8 [12].

**Figure 9.**
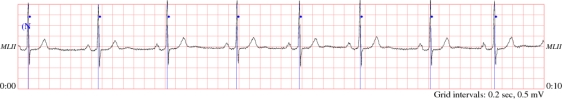
The original ECG data graph.

The ECG data are loaded on the PDA side, transferred to float type and recorded in the RAM. This sampling data was sampled every three milliseconds, and there are 333 ECG data samples per second. The original ECG graph is shown in Figure 9 [13].

The format of body temperature is same as the ECG data and is randomly generated floating point in the temperature range from 36.3 to 37.2 °C.

### 4.2 Monitoring System Implementation

**Figure 10.**
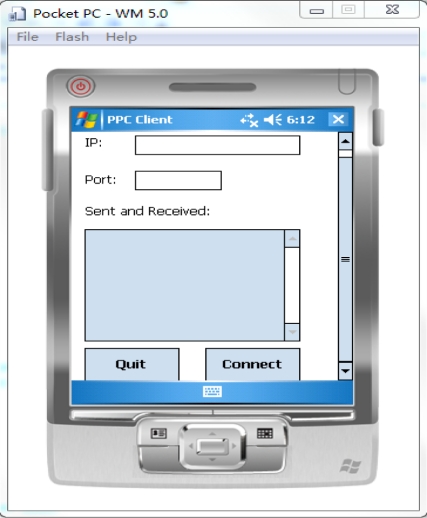
GUI on the PDA simulator.

**Figure 11.**
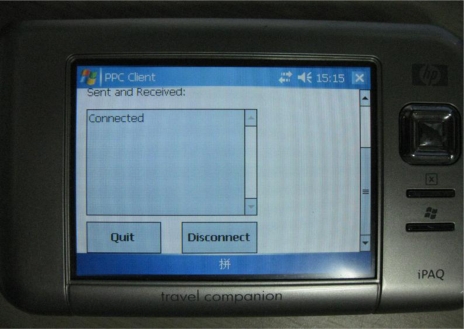
The UI shows that PDA has connected to server.

The user interface of the PDA is shown in Figure 10. The program loads the test sampling data when it is launched, and the user can input the IP address and port number of the server. The connected user interface is shown in Figure 11.

The PDA connects to port 1025 and 1026 of the server, and server receives ECG and temperature data at both of two ports. As the PDA sends 333 ECG sampling data per second, it must send 1,332 bytes per second to server, due to the data being float type.

**Figure 12.**
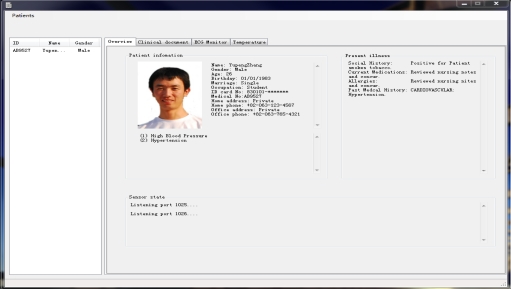
The user interface of the monitoring program.

The user interface of monitoring program is shown in Figure 12. The monitoring program supports creating user information, and records it as an XML file, conforming to the HL 7 clinic document standard.

The main page of the user interface shows personal information and the illness description. The user list is shown in the listview control on the left, and medical personnel can click the “Patients” button on the menu to add a user and display them in the listview control. The user’s information is then displayed in the “Overview” tab on the tabframe control. Medical personnel also can delete a user selected in the listview control, and the personal information is then removed. The program continually listens to the 1025 and 1026 ports for any client connection, and the connection state is shown in “Overview” tab. The Tabframe control consists of four separate tabs. The “Overview” tab shows the user personal information, illness and connection states and “Clinical document” tab shows the personal information and illness description in XML, conforming to clinic document architecture of the HL 7 standard.

**Figure 13.**
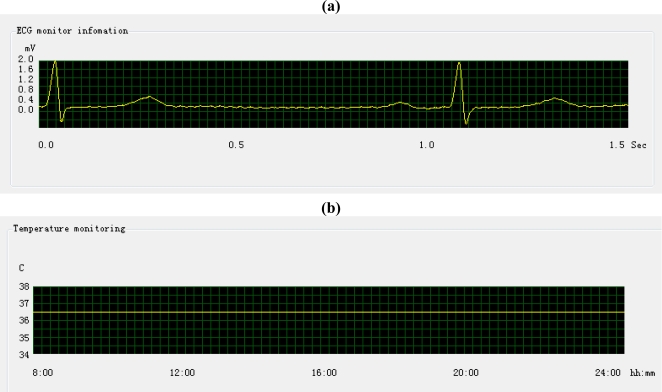
The ECG and temperature monitoring. (a) The ECG monitoring graph; (b) The temperature monitoring graph.

The “ECG monitor” and “Temperature” tab show the graph of ECG and the temperature sampling data. The sampling data come from the PDA, and the monitoring program determines the type of sample data from TEDS, during initial PDA connection. The display monitoring of ECG and temperature data is show an in Figure 13.

The example above shows good patient condition by the sampling data. Note that one square grid in Figure 13 (a) stands for 0.018 s, so the length between two pulses is longer than the same length in Figure 9, due to its higher time precision. Correspondingly, one square grid in Figure 13 (b) represents 30 s.

## 5. Conclusions

This paper proposes the development of a medical information system monitoring for U-Healthcare. Research attention has been given to medical information systems and the increasing need to digitize and exchange medical information. HL7 is a popular medical information exchange standard that is applied in most countries. Due to its specific framework, the HL 7 standard can be readily applied on the personal computer architecture and some mobile devices, but is more difficult with embedded device implementation. The IEEE 1451 is a standard for communications of a transducers and sensors, and it provides a very convenient way to exchange sampling data. The sampling data from sensors conforming to IEEE 1451 can be easily recorded in the documents conforming to the HL 7 standard, and can also be transmitted to other computers with HL 7 messaging protocols proposed, as previously described in this paper. The proposed system focused on monitoring data exchanged between the GUIs and the sensors for the patient and medical personnel. The exchange process was described as: (1) the client informed the server about the property of sampling data by TEDS, which is a part of IEEE 1451 standard, and the server permitted the successive sampling data transmission, and (2) the server recorded sampling data in database, and saved a representative part of graph, which was drawn from the sampling data, as determined by the expertise of medical personnel, in the clinic document. Thus, medical personnel can view the sampling data graph in a clinic document that conforms to CDA specifications in the HL 7 standard.

Privacy is a primary concern and the HL7 standards support clinic document protection to prevent privacy information leakage. The clinic document can only be created by a licensed medical person and can only be modified by the doctor in charge. The clinic document can be viewed by doctor in charge or the person who has been authorized by the doctor in charge. As mentioned in Section 3.1, data are encrypted for transmission by an encryption connection link, such as Secure Sockets Layer (SSL). Reference [13] proposed a Bluetooth security protocol to the Host Controller Interface (HCI), to enhance the transmission safety between the sensor and the mobile computational device. This protocol encrypts the private information following the Advance Encryption Standard (AES), and indentifies the Bluetooth device with the MAC, thereby insuring secure service.

There are some issues that need further consideration, such as the data exchange between two servers using HL 7 messages. A monitoring HL 7 message used to transmit observation data has several segments and the name of each segment is expressed with three words. The header segment is called “MSH”, and the segment used for records sampling data is called “OBS”. The sampling data can be recorded in an OBS segment of XML, or other text type. Further work is recommended to evaluate the implementation of the sampling data exchange among servers with HL 7 messages.

## Figures and Tables

**Table 1.** The TED header structure.

**Field**	**Contents**	**Function**
Length	06	This field is always set to 6, indicating that the header size is 6 bytes.
Type	03	Always 3; indicates that this is TEDS type table
Family	00	Identifies of IEEE 1451 family. This paper conforms to 1451.0, so this field is set to 0.
Class	1 or 3	1 indicates Meta-TEDS and 3 indicates TransducerChannel TEDS.
Tuple Length	Variable	Indicates the size of entail TEDS except header.

**Table 2.** Partial listing of Meta-TEDS.

**Field Name**	**Description**	**Size (bytes)**
TEDSID	TEDS header	6
UUID	Globally Unique Identifier	10
OholeOff	Operational time-out	4 (float)
TestTime	Self-Test time	4 (float)
MaxChan	Number of implemented TranducerChannels	2

**Table 3.** Partial listing of Transducer Channel TEDS.

**Field Name**	**Description**	**Size (bytes)**
TEDSID	TEDS header	6
ChanType	Transducer Channel type. 0 indicates sensor, 1 indicates Actuator, 2 indicates Event sensor.	1
UnitType	Physical Units interpretation enumeration	1
DatModel	Data model	1
SigBits	Significant bit	2
SPeriod	Sampling period (t_sp_)	4 (float)

**Table 4.** The commands descriptions.

**Command ID**	**Command**	**Description**	**Reply expected**	**Directions Mobile Device(md); sensor (sn)**
1	md_QueryMedId	Query the medical ID	Yes	md → sn
2	md_QueryPwd	Query the password	Yes	md → sn
3	md_ConntReq	Connection request	Yes	md → server
4	md_DisconReq	Disconnection request	No	md → server
5	md_ConnMatan	Connection maintain response	No	md → server
6	sr_ConntAprv	Connect request approved	No	server → sn
7	sr_ConntWait	Connect wait in queue	No	server → sn
8	sr_QueryTEDS	Query the TEDS	Yes	server → sn
9	sr_ReadTEDS	Require TEDS contents	Yes	server → sn
10	sr_ChanPara	Channel information	No	server → sn
11	sr_InfoCorm	Confirmed the channels and TEDS	No	server → sn
12	sr_ConnMatan	Connection maintain query	Yes	server → sn

**Table 5.** System environment.

**Monitoring Center Environment**	**PDA Environment**
CPU: Intel Core T8500 Memory: 2,048 MB Language: C# Framework: .NET framework 2.0 IDE: Visual studio 2005 Database: text-based file structure OS: Windows 7 Ultimate	Processor: Samsung SC 32442 at 400 MHz Installed memory (RAM): 64 M Installed memory (ROM): 2 GB Wireless support: WLAN 802.11 b/g, Bluetooth 2.0 OS: Microsoft Windows Mobile 5.0 for Pocket PC, Premium Edition
